# A synthesized olean-28,13β-lactam targets YTHDF1-GLS1 axis to induce ROS-dependent metabolic crisis and cell death in pancreatic adenocarcinoma

**DOI:** 10.1186/s12935-022-02562-6

**Published:** 2022-04-02

**Authors:** Shijia Wu, Yong Ai, Huimin Huang, Guangyu Wu, Shipeng Zhou, Weilong Hong, Percy David Papa Akuetteh, Guihua Jin, Xingling Zhao, Yihua Zhang, Xiaolong Zhang, Linhua Lan

**Affiliations:** 1 Key Laboratory of Diagnosis and Treatment of Severe Hepato-Pancreatic Diseases of Zhejiang Province, The First Affiliated Hospital of Wenzhou Medical University, Wenzhou, 325000 People’s Republic of China; 2 Department of Pharmacology, University of Maryland, Baltimore, USA; 3 State Key Laboratory of Natural Medicines, China Pharmaceutical University, Nanjing, 210009 People’s Republic of China; 4 Jiangsu Key Laboratory of Drug Discovery for Metabolic Diseases, China Pharmaceutical University, Nanjing, 210009 People’s Republic of China; 5 School of Laboratory Medicine and Life Sciences, Wenzhou Medical University, Wenzhou, 325000 People’s Republic of China; 6 Department of Trauma Surgery, The First Affiliated Hospital of Wenzhou Medical University, Wenzhou, 325000 People’s Republic of China; 7 Department of Anesthesiology, Critical Care and Pain Medicine, The Second Affiliated Hospital and Yuying Children’s Hospital of WenZhou Medical University, 109 Xueyuan Western Road, Wenzhou, Zhejiang 325027 People’s Republic of China

**Keywords:** Oleanolic acid, Metabolic crisis, Glutaminolysis, YTHDF1, Redox homeostasis

## Abstract

**Background:**

Pancreatic adenocarcinoma (PAAD) is a severe malignant with a 5-year survival rate of approximately 9%. Oleanolic acid is a well-known natural triterpenoid which exhibits pharmacological activities. We previously synthesized a series of oleanolic acid derivatives and evaluated the tumor-suppressive activity of olean-28,13β-lactam (B28) in prostate cancer. However, the detailed mechanism remains to be understood.

**Methods:**

The anti-tumor activity of B28 in PAAD was confirmed by RTCA, colony formation assay and flow cytometry. GO and KEGG enrichment analyses were performed to analyze the differentially expressed genes (DEGs) obtained by RNA sequencing. The effects of B28 on cell bioenergetics were evaluated by seahorse analyzer. Lenti-virus packaged plasmids were performed to knockdown or overexpress target genes. Alteration of mitochondrial membrane potential, ROS and GSH/GSSG were measured by corresponding detection kits according to the manufacturer's protocol.

**Results:**

We evaluated and confirmed the promising anti-tumor activity of B28 in vitro. RNA-seq profile indicated that multiple metabolic pathways were interrupted in B28 treated PAAD cells. Next, we demonstrated that B28 induces cellular bioenergetics crisis to inhibit PAAD cells growth and induce cell death. We further validated that cell cycle arrest, inhibition of cell growth, cell apoptosis and cell bioenergetics disruption were functionally rescued by ROS scavenger NAC. Mechanistically, we found glutamine metabolism was inhibited due to B28 administration. Moreover, we validated that down-regulation of GLS1 contributes to ROS generation and bioenergetics interruption induced by B28. Furthermore, we elucidated that YTHDF1-GLS1 axis is the potential downstream target of B28 to induce PAAD cell metabolic crisis and cell death. Finally, we also confirmed the anti-tumor activity of B28 in vivo.

**Conclusions:**

Current study demonstrates B28 disrupts YTDFH1-GLS1 axis to induce ROS-dependent cell bioenergetics crisis and cell death which finally suppress PAAD cell growth, indicating that this synthesized olean-28,13β-lactam maybe a potent agent for PAAD intervention.

**Supplementary Information:**

The online version contains supplementary material available at 10.1186/s12935-022-02562-6.

## Background

Pancreatic adenocarcinoma (PAAD) is the most malignant tumor in the digestive system, and is ranked as the seventh leading cause of cancer death in the world by Global Cancer Epidemic Statistics (GLOBOCAN) [1–3]. The incidence of PAAD tumors in China continues to rise, accounting for eighth of malignant tumors [4]. The etiology of PAAD is unclear. Due to strong local spread and distant invasion capabilities, outcome of PAAD patients remains severely poor. The incidence and mortality of PAAD have increased significantly in recent years. The average survival rate is less than 9% [5]. Surgery is currently the main method for PAAD therapy. However, due to the lack of early diagnosis indicators, most patients are in advanced stage with tumor metastasis when diagnosed with PAAD. Additionally, the high postoperative recurrence rate and poor prognosis result to certain clinical limitations of surgery treatment. Traditional adjuvant chemotherapy drugs such as fluorouracil (5-Fu), gemcitabine, and capecitabine have limited efficacy due to poor drug tolerance, severe toxic and side effects which could not significantly improve the survival time of PAAD patients and reduce the recurrence rate [6]. Therefore, it is urgent to find more effective drugs for the treatment of PAAD.

Oleanolic acid (OA) is a well-known natural triterpenoid which has important pharmacological activities in anti-oxidant, anti-hypotensive, hypoglycemic effect, anti-tumor and anti-atherosclerosis. Oleanolic Acid Methyl (CDDO-Me) is a synthetic derivative of OA, which has more potent anti-inflammatory and anti-tumor effects than OA [7]. Low concentrations of CDDO-Me can regulate redox homeostasis and alleviate inflammatory response; higher concentrations of CDDO-Me can selectively inhibit tumor cell growth and induce apoptosis [8]. CDDO-Me has been used in clinical trials to treat diseases such as cancer and chronic kidney disease [9]. Clinical trials have proven that CDDO-Me has a long half-life, good bioavailability and tumor suppressive effect. Previous study has proved that CDDO-Me was well tolerated, but still has unavoidable side effects such as cardiovascular harm [9]. To overcome the unavoidable side effects of CDDO-Me, multiple synthetic OA derivatives (SOADs) have been synthesized [10, 11]. These SOADs have stronger anti-tumor and anti-inflammatory properties via inducing cell apoptosis and activating cyto-protective signal pathways [11, 12]. Our group has also synthesized a series of SOADs and evaluated the potential anti-tumor activity in vitro and in vivo. We found olean-28,13β-lactam (B28) was more efficient to induce cell cycle arrest, cell apoptosis and inactivate Akt/mTOR signal pathway in prostate cancer comparable to CDDO-Me. Additionally, B28 was more stable in rat plasma and human liver microsomes with little inhibition of hERG channel [13]. However, the detailed mechanism of B28 in suppressing cancer cell growth remains largely unknown. Our data showed B28 exhibited strong tumor suppressive activity in PAAD in vitro. Moreover, transcriptomics data identified multiple cell metabolism processes were interrupted by B28 especially in carbon metabolism, oxidative phosphorylation (OXPHOS), glycolysis and Tricarboxylic acid cycle (TCA cycle).

It is well demonstrated that metabolic reprogramming is an important hallmark of cancer cell progression. Cancer cells commonly reprogram metabolic pathways such as aerobic glycolysis, TCA cycle, and glutamine metabolism to meet energy requirements for rapid proliferation, invasion, and metastasis. Metabolic reprogramming also provides sufficient precursors for cancer growth and proliferation for the synthesis of biological macromolecules such as lipids, proteins and nucleic acids [14]. Metabolomics is emerging to provide new insight for cancer diagnosis which highlights crucial roles of cancer metabolic reprogramming in cancer [15]. Our previous studies also validated that targeting cellular bioenergetics is an efficient strategy to inhibit PAAD in vitro and in vivo [16, 17]. Thus, according to the effect of B28 on multiple cell metabolic pathways, we speculated that whether B28 could inhibit PAAD cell growth through disrupting PAAD cell metabolism. Therefore, in current study, we analyzed mitochondrial respiration and aerobic glycolysis to evaluate the effects of B28 on disrupting cellular bioenergetics and investigate potential mechanism to address this hypothesis.

## Materials and methods

### Reagents and antibodies

Crystal violet stain solution (C0121), Cell Cycle Analysis Kit (C1052), Reactive Oxygen Species Assay Kit (S0033S), Mitochondrial membrane potential assay kit with JC-1 (C2003S), Cell Counting Kit-8 (C0039), *N*-acetyl-l-cysteine (ST1546), DAPI Staining Solution (C1005), GSH and GSSG Assay Kit (S0053), Caspase 3 Activity Assay Kit (C1115) were purchased from Beyotime Biotech (Jiangsu, China). Protease inhibitor MG-132 (S2619) was purchased from Selleck (Houston, Texas, U.S.). XF Cell Mito Stress Test Kit (103010-100) and XF Glycolysis Stress Test Kit (103017-100) were purchased from Agilent Technologies (Beijing, China). MitoTracker™ Red FM (M22425), BCA protein concentration detection kit (23225) and Pierce ECL Western blotting substrate kit (32132X3) were obtained from Thermo Fisher Scientific, Inc. (Waltham, MA, USA). FITC Annexin V Apoptosis Detection Kit I (556547) was purchased from BD Biosciences (Franklin Lake, New Jersey, U.S.) and Annexin V-FITC/7-AAD Apoptosis Detection Kit (E-CK-A212) was purchased from Elabscience Biotechnology (Wuhan, China), respectively. 2-NBDG (186689-07-6) was obtained from Anjiekai Biological Medicine Technology (Wuhan, China). Lactic Acid assay kit (K607-100) was used from Biovision (California, USA). The detailed information of antibodies used in this study was summarized in Additional file 6: Table S1.

### Cell line and cell culture

PAAD cell lines PANC-1, Miapaca-2 and CFPAC-1 cells were obtained from the Cell Bank of the Chinese Academy of Sciences (Shanghai, China). Cells were cultured in DMEM medium (C11995500BT, GBICO, Invitrogen, California, U.S.) supplemented with 10% fetal bovine serum (10099141, Gibco™, Invitrogen, California, U.S.) and antibiotics (100 U/mL penicillin and 100 μg/mL streptomycin, C0222, beyotime, Jiangsu, China) at 37 °C, 5% CO_2_.

### Real time cell growth determination by RTCA

50 μL cell culture medium was added in E-Plate 16 PET and baseline was calibrated. Then, PAAD cells were seeded in E-Plate 16 PET at a density of 5 × 10^3^/well and cultured at 37 °C, 5% CO_2_ incubator overnight. Next day, cell were treated with vehicle or B28 (0.125, 0.25, 0.5, 1 μM, respectively), real-time cell index was measured and recorded by xCELLigence RTCA DP.

### Colony formation assay

PANC-1 (800 cells/well), Miapaca-2 (1000 cells/well) and CFPAC-1 (800 cells/well) cells were plated in 6-well plates and cultured at 37 °C, 5% CO_2_ for 48 h. Then, cells were treated with or without B28 (0.25, 0.5, 1 μM), cell culture medium was replaced every 3 days. After 10–14 days, cells were washed with PBS for two times and fixed in methanol. Subsequently, cells were stained with 0.2% crystal violet stain solution for 15 min at room temperature. Cells were washed with PBS to remove residual crystal violet stain solution. Cell culture plates were capture captured and colony area was quantified by Image Plus software.

### Cell cycle distribution analysis

PANC-1 cells were cultured with or without B28 (0.25, 0.5, 1 μM) at 37 °C, 5% CO_2_ overnight. Then, cells were digested by 0.25% EDTA-trypsin and collected. Collected cells were washed with PBS for two times and fixed by 80% ethanol at 4 °C overnight. Next, cell samples were stained with PI dye solution at room temperature followed by flow cytometry analysis.

### Cell apoptosis detection

PANC-1 cells were seeded into 6-cm dishes and treated with or without B28 (0.25, 0.5, 1 μM) for 24 h. Cells were collected and washed with ice-cold PBS for two times. Collected cell pellet was re-suspended with Annexin V + PI mixture and incubated at room temperature for 20 min, samples were protected from light. 200 μL 1× binding buffer solution was added into each tube. Next, apoptosis analysis of indicated samples were determined and analyzed by flow cytometry and flow Jo software, respectively.

### Caspase 3 activity detection assay

Vehicle and B28 pretreated PANC-1 and CFPAC-1 cells were digested with trypsin (0.25% EDTA), cells were collected by centrifuging at 600×*g*, 4 °C for 5 min. Collected cell pellet was washed with PBS for one time. Next, lysis buffer was added at a ratio of 100 μL/2 × 10^6^ cell and placed in ice for 15 min. Then, samples were centrifuged at 16,000×*g*, 4 °C for 15 min. The supernatant was transferred into a new tube and mixed well with 10 μL Ac-DEVD-pNA (2 mM). The mixture was incubated at 37 °C for 2 h. The standard curve of pNA (0, 10, 20, 50, 100 and 200 μM) was measured according to the OD value at 405 nm. OD values at 405 nm of blank and samples were also measured. The relative activity of caspase 3 was calculated by the standard curve of pNA. One unit of caspase 3 is the amount of enzyme that cleaves 1.0 nmol of the colorimetric substrate Ac-DEVD-pNA per hour at 37 °C under saturated substrate concentrations [18]. Then, the calculated activity of caspase 3 in vehicle samples was normalized to 1. The data was presented as fold change of caspase 3 activity in B28 treated samples divided by that in vehicle treated samples.

### RNA-seq for transcriptomics

PANC-1 cells were treated with or without B28 (1 μM) for 24 h. Then, cells were collected and mixed with Trizol (Invitrogen, Carlsbad, CA, US). RNA purity was detected by Nano Photometer spectrophotometer and RNA integrity was measured by Agilent 2100 bio-analyzer. RNA sequencing data and report were completed by Novo Gene Technology Co., Ltd. (Beijing, China). Gene function enrichment analysis was based on Gene Ontology Resource (GO): http://geneontology.org/, Kyoto Encyclopedia of Genes and Genomes (KEGG): https://www.genome.jp/kegg/, Differentially expressed genes (DEGs) in vehicle and B28 treated samples were selected by the packages “clusterProfiler”, “ggplot2”, “org.Hs.eg.db”, and “enrichplot”.

### Oxygen consumption rate (OCR) and extracellular acidification rate (ECAR) detection

In brief, PANC-1 and CFPAC-1 cells were seeded into 96-well seahorse cell culture plate and cultured at 37 °C, 5% CO_2_ incubator overnight. Next, cells were treated with vehicle or B28 (0.5, 1 μM) for 6 h. Then, OCR and ECAR were measured by seahorse XFe96 bioenergetics analyzer according to the manufacturer’s protocol. Preparation of assay medium OCR detection: 49 mL seahorse basal medium, 1 mL pyruvate sodium and 0.225 g d-glucose, pH 7.4. Cells were rinsed with 110 μL assay medium for twice and then 175 μL assay medium was added into each well. Next, oligomycin, FCCP, antimycin A and rotenone were sequentially injected according to the protocol. The final concentration of above compounds was 1 μM. Preparation of assay medium for ECAR detection: 0.0164 g l-glutamine was dissolved in 50 mL seahorse basal medium,, pH7.4. Cells were rinsed with 110 μL assay medium for twice and then 175 μL assay medium was added into each well. Next, d-glucose, oligomycin, 2-DG were sequentially injected, and the final concentration of above compounds was as follows: 10 mM, 1 μM and 100 mM, respectively. All the samples have at least five replicates. The protocol of OCR and EACR detection in other groups is similar to described above.

### Western blot analysis

Expression of protein expression was detected by Western blot. Briefly, total cell protein was extracted by triton X-100 lysis buffer and protein concentration was determined by BCA protein concentration detection kit. Total 20 μg/well protein was loaded into 10% or 12% SDS-PAGE gels. Separated proteins were then transferred to 0.22 μM PVDF membrane at 100 V for 80 min. Next, membranes were blocked in Tris-buffered saline (TBS) containing 5% non-fat dry milk and 0.1% Tween 20 (TBS-T) at room temperature for 1.5 h. Then, membranes were washed with TBS-T for 2 min to remove residual blocking solution and incubated with the primary antibodies at 4 °C overnight. Next day, membranes were washed with TBS-T for 3 times, 5 min each time. Next, the blots were incubated with the appropriate horseradish peroxidase-conjugated secondary antibody for 1 h. Finally, membranes were incubated with ECL solution and signal was captured by Analytik Jena Chem Studioplus85. The antibodies used in this study were summarized in Additional file 6: Table S1.

### Mitochondrial visualization by Mito-Tracker Red

PANC-1 cells were seeded into cover slip and cultured with or without B28 for 12 h at 37 °C, 5% CO_2_ incubator overnight. Next day, cells were washed with PBS for two times and fixed by 4%PFA for 15 min. Cells were washed with PBS and incubated with Mito-Tracker Red (80 nM) for 40 min. Then, cells were washed with PBS for 3 × 5 min and incubated with DAPI (10 μg/mL) for 8 min at room temperature. Finally, cells were washed with PBS and fluorescence signal was captured by confocal laser scanning microscope.

### Total reactive oxygen species (ROS) determination

PANC-1 cells were treated with vehicle and B28 (0.25, 0.5, 1 μM) for 24 h, total ROS was measured by flow cytometry. In brief, cells were digested and collected in 1.5 mL tubes. Cell pellet was washed with FBS-free DMEM basal medium for two times. Then, cell samples were further incubated with DCFH-DA (10 μM) for 20 min in dark. Next, stained cells were washed with FBS-free DMEM for three times to remove residual free DCFH-DA probe. Then, cell samples were mixed well with 300 μL FBS-free DMEM basal medium followed by flow cytometry analysis.

### Mitochondrial membrane potential measurement

Vehicle and B28 treated PANC-1 cells were collected and washed with PBS for two times. Collected cells were centrifuged at 1000 rpm for 5 min. Then, cell pellet was incubated with JC-1 probe at 37 °C for 20 min and then washed with 1× dye solution buffer for two times. Finally, fluorescence signal of JC-1 monomer and J-aggregates were measured by flow cytometry analysis.

### Real-time PCR to measure GLS1 mRNA transcripts

Total RNA was extracted from cells using Trizol (Invitrogen, Carlsbad, CA, US). The primers of target genes are listed as follows: human GLS1: forward; 5′-CAGAAGGCACAGACATGGTTGG-3′ and reverse 5′-GGCAGAAACCACCATTAGCCAG-3′; human β-actin: forward: 5′-TCTGGCACCACACCTTCTAC-3′ and reverse 5′-GATA GCACAGCCTGGATAGC-3′. PCR reaction conditions were as follows: denaturation at 94 °C, 3 min; 40 cycles of 94 °C for 30 s, 59 °C for 30 s, and 72 °C for 45 s to amplify indicated genes.

### Cell viability measurement by CCK-8 assay

For series metabolites addition assay: PANC-1 cells were treated with B28 (1 μM) or B28 (1 μM) plus α-KG (2 mM), Isocitric acid (2 mM), Citric acid (2 mM), Succinic acid (2 mM), Malate (2 mM), Fumaric acid (2.5 mM), Oxalacetic acid (5 mM), Glutamate (2.5 mM), Glutamine (2.5 mM) or NAC (5 mM), respectively. Cells were cultured at 37 °C, 5% CO_2_ incubator for 24 h. Then, 10 μL CCK-8 working solution was added in each well and cell plate was incubated at 37 °C, 5% CO_2_ incubator for another 2 h. Finally, OD values at 450 nm were measured by micro-plate reader. Relative cell viability was represented as OD_450_. For cell proliferation detection, shCont and GLS1 knockdown PAAD cell lines were seeded into 96 well plates at a density of 4 × 10^3^ cells/well. Then, CCK-8 working solution was added into each well and OD value at 450 nm was measured at 24, 48, 72, 96 h. Cell proliferation curve was graphed according to the OD_450_ values.

### Lenti-virus package and RNA interfere

Lenti-virus package: HEK293 cells were cultured and co-transfected with packaging plasmid psPAX2 (2 μg), envelope plasmid pMD2G (1 μg) and target plasmids GLS1 shRNA (2 μg) or YTHDF1 shRNA (2 μg) or YTHDF1 overexpressed plasmid (2 μg). Then, cell culture medium was collected at 24 h, 48 h and 72 h post-transfection. Collected cell culture medium was filtered through a 0.45 μM filter. Virus solution was stored at − 80 °C for further use. RNA interference: PANC-1 and CFPAC-1 cells were transfected with lenti-virus packaged indicated plasmids. Successfully transfected cells were screened by puromycin and verified by western blot. Detailed information of indicated plasmids was summarized in Additional file 7: Table S2.

### Anti-tumor activity determination of B28 in vivo

Xenograft nude mice assay was in accordance with the guidelines of the Institutional Animal Care and Use Committee, University Laboratory Animal Research of Wenzhou Medical University. PANC-1 cells (6 × 10^6^) were subcutaneously injected into the left flank of 5-week-old mice (Male, n = 10). The mice were randomly divided into two groups (5 mice per group). Then, the mice of one group were administrated with B28 (6 mg/kg) for each and another was intraperitoneally injected with 0.9% NaCl. Tumor size was monitored 2 days in 3 weeks. The volume of tumor size was calculated according to the formula: 1/2 × L × W^2^. For the Euthanasia, nude mice were intraperitoneally injected with sodium pentobarbital (dose: 200 mg/kg). After confirming that the nude mice have died, the tumor sections were resectioned, weighted and captured.

### Statistical analysis

Statistical data analyses was carried out and graphs were generated by SPSS 16.0 statistical software package (SPSS Inc., Chicago, IL, USA) and Graph Pad Prism 5.0 software (Graph Pad Software, Inc., La Jolla, CA, USA), respectively. Differences between groups were analyzed by a two-sided student’s t test. *p < 0.05, **p < 0.01, ***p < 0.001, n.s. represents no significance.

## Results

### Olean-28,13β-lactam (B28) suppresses PAAD cell growth and induces PARP-caspase 3 dependent cell apoptosis

**Fig. 1:**
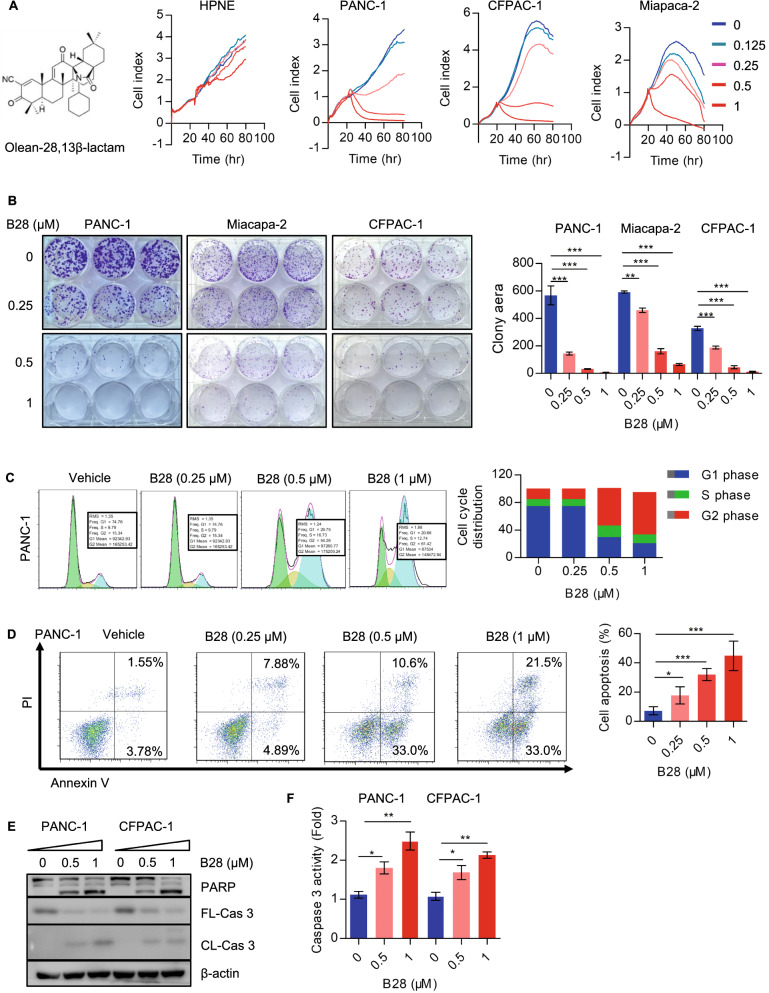
Olean-28,13β-lactam (B28) inhibits PAAD cell growth in vitro and in vivo. **A** Chemical structure of oleanolic acid, oleanolic acid methyl and olean-28,13β-lactam (B28). PANC-1, CFPAC-1 and Miapaca-2 cells were treated with vehicle and B28 (0.125, 0.25, 0.5, 1 μM), real time cell index was measure by RTCA. **B** Colony formation results of vehicle and B28 treated PAAD cells. **C** PAAD cells were treated with or without B28 for 24 h, cell cycle distribution was detected and analyzed by flow cytometry. **D** PAAD cells were treated with B28 for 24 h and subjected to Annexin V/PI apoptosis detection kit. Apoptotic cells were visualized and analyzed by flow cytometry and flow Jo software, respectively. **E** PANC-1 cells were treated with or without B28 (0.5, 1 μM) for 24 h and subjected to western blot analysis with cell apoptosis associated antibodies. **F** Caspase 3 activity of vehicle and B28 treated PAAD cells. Data are presented as mean ± SD, *p < 0.05, **p < 0.01, ***p < 0.001

In our previous work, we have synthesized a novel derivative of Oleanolic Acid Methyl named Olean-28,13β-lactam (B28) and evaluated promising anti-tumor activity in prostate cancer. We also validated that B28 significantly inactivates Akt/mTOR signal and induces caspase-3 dependent cell apoptosis [13]. Our data showed B28 could also effectively suppress PAAD cell growth which indicated potential anti-tumor activity of B28 in PAAD. Importantly, we found no significant inhibitory effect of B28 on normal pancreatic cell HPNE (Fig. 1A). To further confirm the inhibitory activity of B28 on PAAD cell growth, we evaluated cell growth of vehicle and B28 treated cells. Colony formation assay data showed cell growth was dramatically inhibited in B28 dose-dependent manner (Fig. 1B). Additionally, we found more G2 phase distribution was accumulated which indicated cell cycle arrest in response to B28 (Fig. 1C). Simultaneously, more apoptotic cells were found in B28 treated cells (Fig. 1D). To further confirm the role of B28 in cell apoptosis initiation, cell apoptosis executors were measured. We found B28 addition increased cleavage of PARP and caspase-3 (Fig. 1E). Moreover, caspase 3 activity in vehicle and B28 treated cells were further detected. We found caspase 3 activity was significantly increased in B28 treated cells (Fig. 1F).

### B28 disrupts multiple crucial processes in PAAD cells

**Fig. 2:**
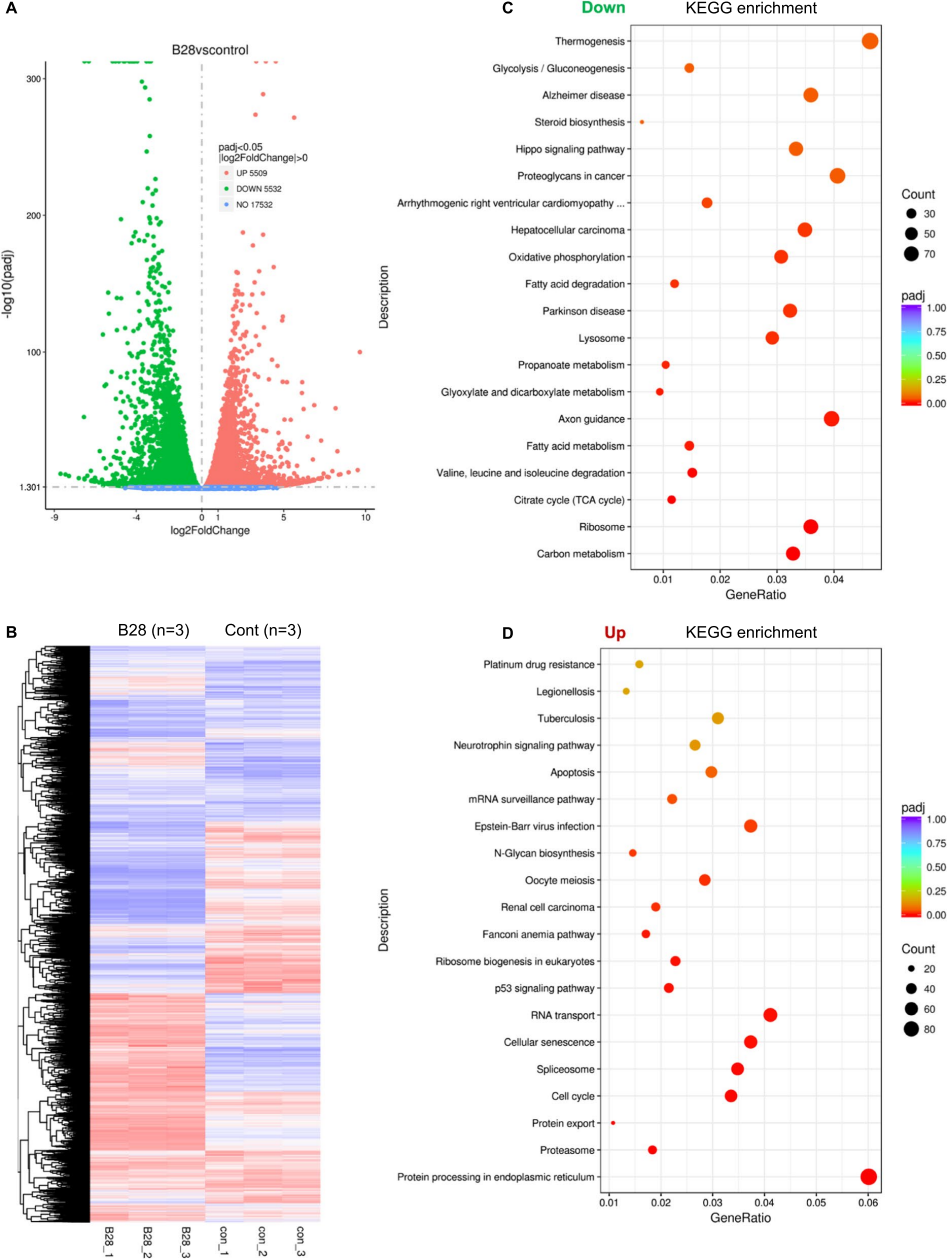
B28 interrupts multiple crucial biological processes in PAAD. **A** PANC-1 cell were treated with or without B28 for 24 h, total RNA of cell samples were extracted and subjected to RNA-seq. Statistical analysis of variance in vehicle and B28 treated groups was plotted as volcano map. **B** Bioinformatics analysis combined with cluster analysis of gene transcription in vehicle and B28 treated cells. **C** Enrichment analysis of down-regulated and up-regulated differentially expressed genes (DEGs) based on KEGG database

To understand molecular mechanism of B28 in suppressing PAAD cell growth, total RNA of vehicle and B28 treated samples were extracted and subjected to RNA-seq analysis. Based on the RNA-seq data, we found 5509 genes were up-regulated and 5532 genes were down-regulated, 17,532 genes were not significantly changed (Fig. 2A, B, Additional files 1 and 2). To understand biological pathways which were modulated by B28 addition, cluster analysis were performed to visualize changes of genes and pathways. Based on the KEGG database, we found down-regulated genes mainly belong to multiple cell metabolism pathways such as carbon metabolism, TCA cycle, propanoate metabolism, fatty acid degradation and glycoxylate and dicarboxylate metabolism as well as OXPHOS. Meanwhile, molecules participating in protein processing in endoplasmic reticulum, RNA transport, and cellular senescence were notably increased in B28 treated cells. Importantly, we found genes which participate in apoptosis pathway, p53 signaling pathway with observations of cell cycle were also increased which was consistent with cell cycle arrest and cell growth inhibition in B28 treated cell samples (Fig. 2C, D, Additional file 3, Additional file 8: Fig. S1). Furthermore, we found TCA cycle, monocarboxylic acid metabolic process, co-factor/co-enzyme metabolic processes and fatty acid metabolic processes were notably reduced in B28 treated samples. We also found genes participating in response to endoplasmic reticulum stress and response to unfolded protein processes were up-regulated which was consistent with the data of KEGG enrichment (Additional file 4, Additional file 8: Fig. S2). These alterations of genes in crucial cell processes were further confirmed by reactome analysis (Additional file 8: Fig. S3). These transcriptomics data summarized and figured out crucial processes which were regulated by B28. Among these pathways, we found that multiple cell metabolic processes were affected by B28 treatment.

### B28 induces mitochondrial dysfunction and energy restriction

**Fig. 3:**
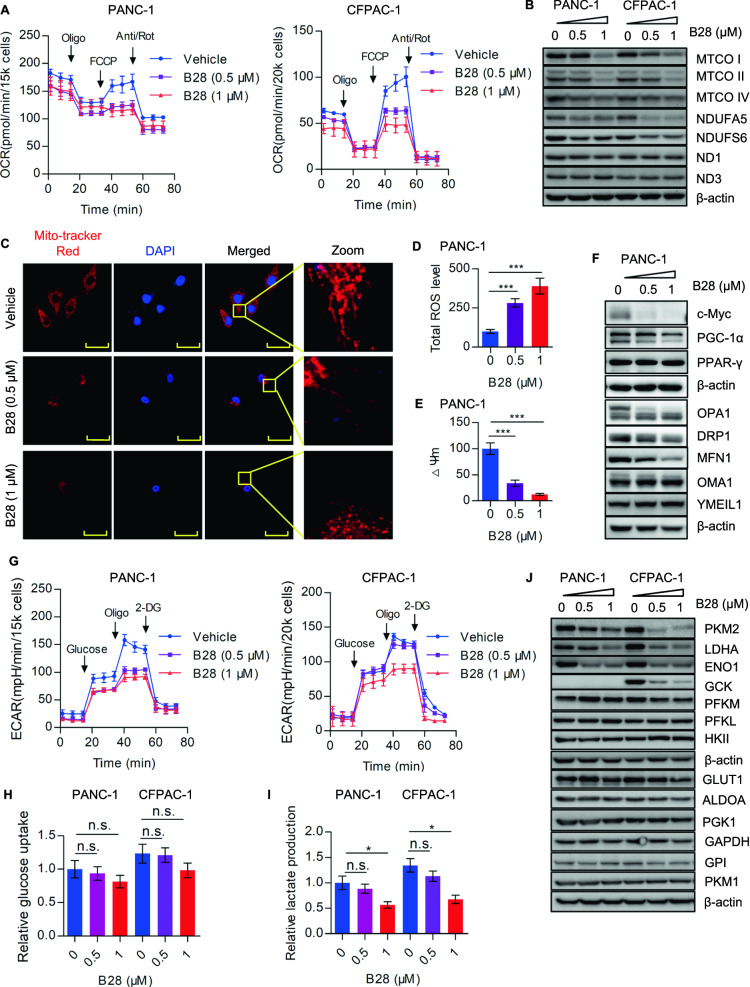
B28 dramatically disturbs cellular bioenergetics. **A** PANC-1 and CFPAC-1 cells were treated with or without B28 (0.5, 1 μM), Overall OCR curves of vehicle and B28 treated cells were measured by seahorse XFe96 bioenergetics analyzer. **B** PANC-1 and CFPAC-1 cells were cultured with or without B28 for 24 h, followed by western blot analysis to detect indicated subunits of mitochondrial respiratory complexes. **C** PANC-1 cells were cultured with or without B28 for 12 h, cells were fixed and stained with Mito-Tracker and DAPI. Signal of mitochondria and nucleus was visualized and captured by confocal laser scanning microscope. **D** Total ROS level in vehicle and B28 treated cells were measured by DCFH-DA ROS detection kit and analyzed by flow cytometry. **E** Mitochondrial membrane potential alteration in vehicle and B28 treated PANC-1 cells were measured by JC-1 mitochondrial membrane potential detection kit and analyzed by flow cytometry, respectively. **F** Vehicle and B28 treated PANC-1 cells were collected and subjected to western blot analysis with indicated antibodies. **G** Overall ECAR curves of vehicle and B28 treated PANC-1 and CFPAC-1 cells were measured by seahorse XFe96 bioenergetics analyzer. **H**, **I** Relative glucose uptake and relative lactate production in vehicle and B28 treated cells, respectively. **J** PANC-1 and CFPAC-1 cells were cultured with or without B28 for 24 h, cells were collected and followed by western blot analysis with primary antibodies against glycolytic catalytic enzymes. Data are shown as Mean ± SD. *p < 0.05, ***p < 0.001, n.s. represents no significance

Next, seahorse XFe96 bioenergetics analyzer was performed to measure overall oxygen consumption rate (OCR) in vehicle and B28 treated PANC-1 and CFPAC-1 cells. We found real-time overall OCR was reduced in B28 dose dependent manner (Fig. 3A). Moreover, basal respiration, maximal respiration, ATP production associated OCR and spare respiratory capacity (%) were decreased upon B28 addition (Additional file 8: Fig. S4A). However, no significance was found in proton leak and non-mitochondrial oxygen consumption rate (Additional file 8: Fig. S4B). Consistently, we found MTCO I, MTCO II, MTCO III and NDUFS6 were reduced in B28 treated cell samples, while ND1 and ND3 were not significantly changed (Fig. 3B). To further visualize mitochondrial morphologic change in vehicle and B28 treated cells, cellular mitochondria was stained by Mito-Tracker Red and captured by confocal laser scanning microscope. More fragmented mitochondria were found in B28 treated cells and fluorescence signal of mitochondria was weaker than vehicle treated cells (Fig. 3C). It has been well demonstrated that mitochondria are the major source of ROS generation, disruption of mitochondria homeostasis commonly enhances ROS production which increases cellular oxidative damage [19]. Thus, we speculated whether B28 could induce mitochondrial dysfunction. To clarify this hypothesis, total cellular ROS was measured and data showed ROS level was increased in B28 dose dependent manner (Fig. 3D). Furthermore, we found mitochondrial membrane potential was also decreased by B28 (Fig. 3E). We next measured protein expression of mitochondrial biogenesis and dynamics associated molecules. Data showed molecules control mitochondrial biogenesis as c-Myc and PGC1-α were both decreased, while PPAR-γ was not significantly changed. Meanwhile, we found OPA1, a crucial mitochondrial dynamics associated protein, was cleaved which indicated mitochondrial dysfunction. Additionally, mitochondrial fission protein DRP1 was also decreased in B28 treated cells. Interestingly, we found mitochondrial fusion protein MFN1 was reduced in response to B28, and no significance of OMA1 and YMEIL was found (Fig. 3F). These data indicated that both mito-fusion and fission were repressed in B28 treated cells which would be important for B28 induced cell apoptosis.

**Fig. 4:**
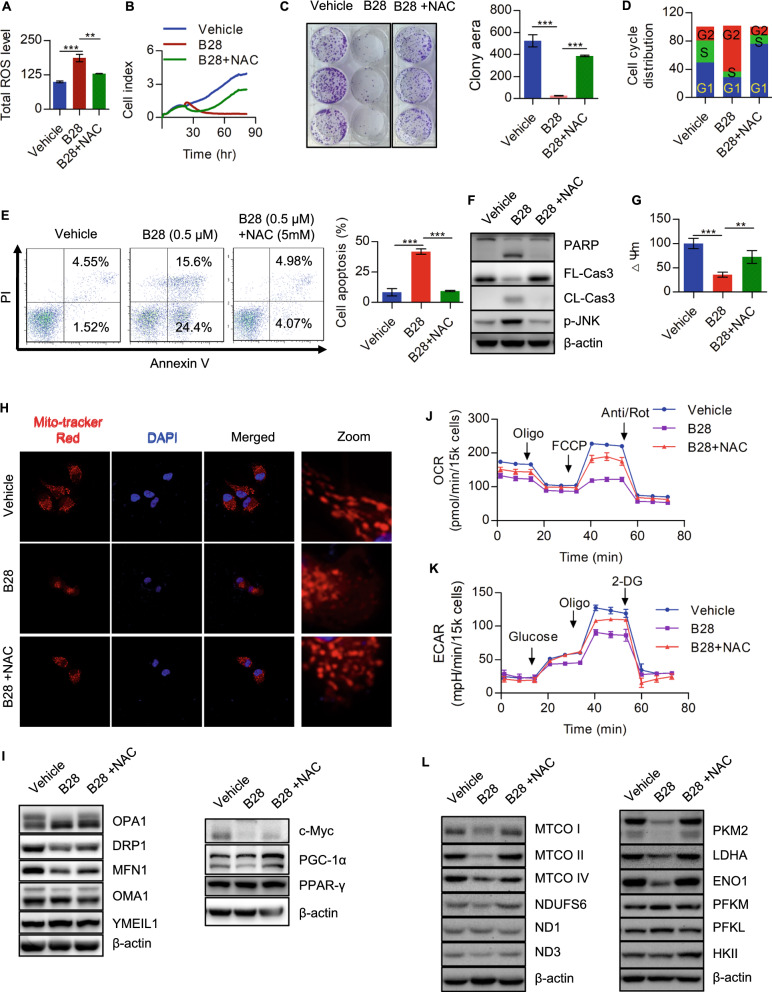
B28 induce cell apoptosis and bioenergetics dysfunction dependent on excessive ROS generation. **A** PANC-1 cells were pretreated with ROS scavenger NAC followed by B28 addition for another 24 h, total ROS level was measured and analyzed by flow cytometry. **B** Cell growth curves of vehicle, B28 and B28 plus NAC treated cells. **C** Colony images and quantitative analysis of vehicle, B28 and B28 + NAC treated PANC-1 cells. **D** Cell cycle distribution analysis of indicated groups was analyzed by flow cytometry. **E** Cell apoptosis in vehicle, B28 and B28 + NAC treated PANC-1 cells were measured and analyzed by flow cytometry. **F** Vehicle, B28 and B28 + NAC treated PANC-1 cells were collected and subjected to western blot analysis with indicated antibodies. **G** Analysis of mitochondrial membrane potential alteration in vehicle, B28 and B28 + NAC treated PANC-1 cells. H PANC-1 cells were pretreated with NAC followed by B28 addition, cells were fixed and stained with DAPI and Mito-Tracker, fluorescence of mitochondria and nucleus was captured by confocal laser scanning microscope. I PANC-1 cells were cultured with vehicle, B28 or B28 + NAC, collected cell samples were subjected to western blot analysis with indicated antibodies. **J**, **K** Overall OCR and ECAR curves of vehicle, B28 and B28 + NAC treated cells. **L** PANC-1 cells were treated with vehicle, B28 or B28 + NAC for 24 h, cell samples were collected and analyzed by western blot with primary antibodies. Data are shown as Mean ± SD. **p < 0.01, ***p < 0.001, n.s. represents no significance

Previous studies have demonstrated that glycolysis process was hyper-activated in tumor cells even under normal oxygen condition, this phenomenon was named ‘Warburg effect’ [20]. Thus, enhanced aerobic glycolysis was considered as a hallmark of cancer cells [21]. Based on the data of mitochondrial respiration, we proposed that whether B28 could also modulate aerobic glycolysis. Here, we found overall ECAR was reduced in PANC-1 and CFPAC-1 cells with B28 treatment (Fig. 3G). This data was consistent with the result of transcriptomics. We also assessed glycolytic associated indexes and found basal glycolysis, glycolysis capacity and glycolysis reverse were more or less decreased due to B28 addition (Additional file 8: Fig. S4C). Moreover, no significant change of glucose uptake was found between two groups, while relative lactate production was reduced within B28 treatment (Figs. 3H and 4I). To further confirm the effects of B28 on aerobic glycolysis, several glycolysis catalytic enzymes were detected. We found PKM2, LDHA and ENO1 were reduced in B28 treated cells, while other indicated enzymes were not significantly changed (Fig. 3J). These data indicate that B28 is effective to repress aerobic glycolysis through down-regulating PKM2-LDHA-ENO1 axis.

### Increased ROS generation caused by B28 results to cell growth inhibition and metabolic crisis

**Fig. 5:**
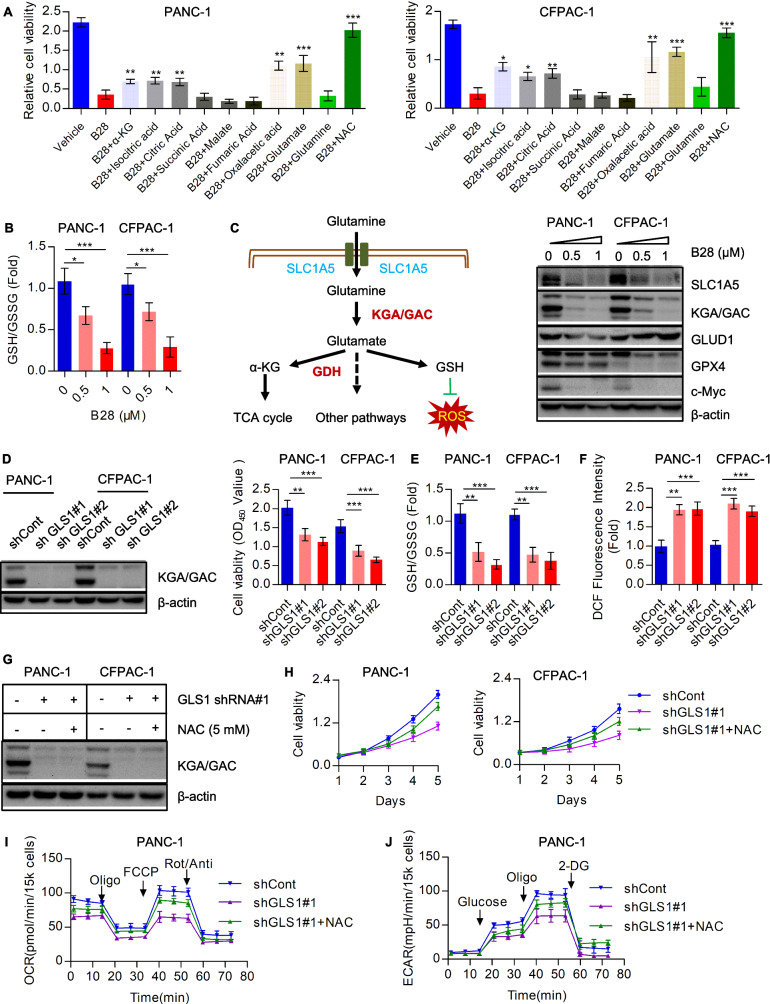
GLS1 is a potential downstream target of B28. **A** PANC-1 and CFPAC-1 cells were treated with B28 or supplemented with multiple metabolites, relative cell viability was measured. **B** Ratio of GSH and GSSG in vehicle and B28 treated cells. **C** Vehicle and B28 treated PAAD cells were collected and subjected to western blot with indicated antibodies. **D** Relative cell viability in shCont and GLS1 knockdown PAAD cells. **E** Ratio of GSH and GSSG in shCont and GLS1 knockdown PAAD cells. **F** Total ROS level in shCont and GLS1 knockdown PAAD cells. **G** Expression of GLS1 in shCont, shGLS1#1 and shGLS1#1 + NAC cells. **H** Cell growth curves of shCont, shGLS1#1 and shGLS1#1 + NAC cells. **I**, **J** Overall OCR and ECAR curves in shCont, shGLS1#1 and shGLS1#1 + NAC cells. Data are shown as Mean ± SD. **p < 0.01, ***p < 0.001

As mentioned above, B28 is effective to interrupt cell metabolism especially in cellular bioenergetics. Importantly, we found B28 induced mitochondrial dysfunction and promoted excessive ROS generation. Therefore, we proposed that whether B28 increased ROS is indispensable for anti-tumor property of B28. To clarify this hypothesis, NAC, a commonly used ROS scavenger, was used to abolish ROS. Data showed NAC was efficient to reduce ROS level in B28 treated cells (Fig. 4A). We further evaluated cell growth in vehicle, B28 and B28 plus NAC cells. We found NAC addition could significantly rescued inhibition of cell growth caused by B28 (Fig. 4B, C). Additionally, we also found cell cycle arrest was dismissed by NAC which was consistent with the data of cell growth (Fig. 4D and Additional file 8: Fig. S5A). Reduced cell apoptosis was found in NAC abolished ROS cells (Fig. 4E). Moreover, we demonstrated cleavage of PARP and caspase-3 was decreased and JNK was also inactivated due to NAC addition (Fig. 4G and Additional file 8: Fig. S5B). Additionally, mitochondrial membrane potential was determined and data showed loss of mitochondrial membrane potential induced by B28 was notably recovered by NAC (Fig. 4G and Additional file 8: Fig. S5B). Furthermore, we found fragmented mitochondria in B28 treated cells were decreased by NAC addition (Fig. 4H). Meanwhile, NAC significantly blocked the reduction of OPA1, DRP1, MFN1, c-Myc and PGC-1α induced by B28 (Fig. 4I). We also found abolishment of excessive ROS by NAC recovered inhibition of mitochondrial respiration and aerobic glycolysis caused by B28 (Fig. 4J, K, Additional file 8: Fig. S5C). Moreover, we found indicated decrease of subunits as MTCO I, MTCO II, MTCOIV and NDUFS6 and glycolysis catalytic enzymes as PKM2, LDHA and ENO1 caused by B28 was also relieved by NAC (Fig. 5L). We suggest that tumor-suppressive activity of B28 probably due to B28 induced excessive ROS generation.

### Down-regulation of GLS1 contributes to oxidative stress and metabolic crisis induced by B28

**Fig. 6:**
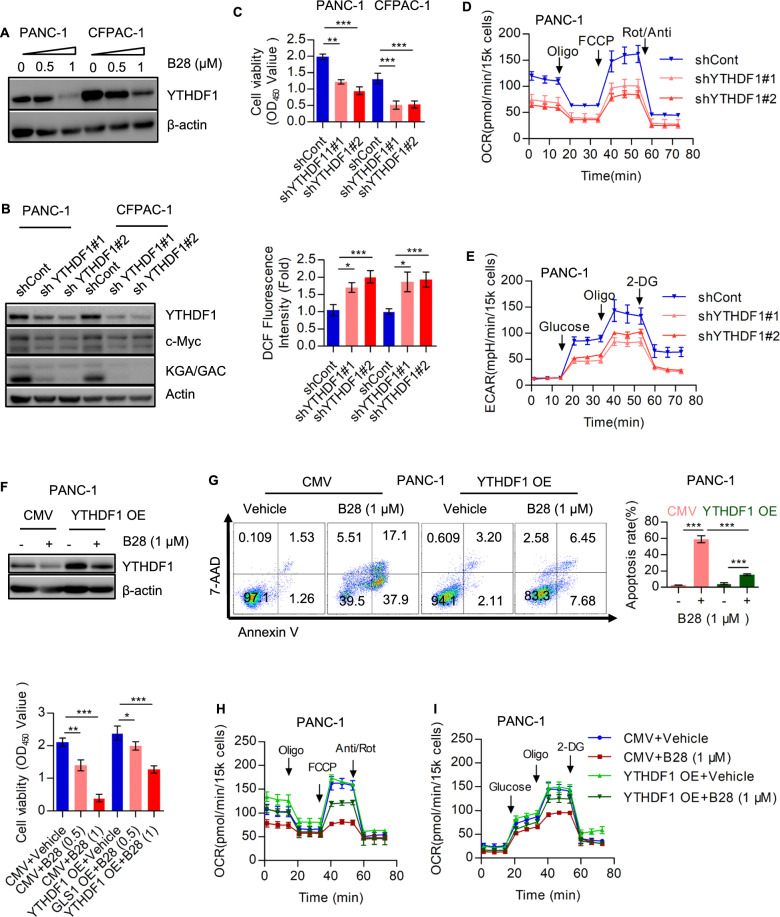
B28 regulates GLS1 expression depends on down-regulation of YTHDF1. **A** YTHDF1 expression in vehicle and B28 treated cell samples. **B** shCont and YTHDF1 depleted cells were subjected to western blot analysis with indicated antibodies. C Relative cell viability and total ROS level in control and YTHDF1 knockdown PAAD cells. **D**, **E** Overall OCR and ECAR curves in control and YTHDF1 depleted cells. **F** Control and YTHDF1 overexpressed cells were treated with or without B28, relative cell viability was measured by CCK-8 assay. **G** Control and YTHDF1 overexpressed cells were cultured with or without B28, cell apoptosis were analyzed by flow cytometry. HandI overall OCR and ECAR curves of CMV + vehicle, CMV + B28, YTHDF1 OE + vehicle and YTHDF1 OE + B28, respectively. Data are shown as Mean ± SD. **p < 0.01, ***p < 0.001

Considering the effect on B28 on mitochondrial respiration, we speculated that whether exogenous metabolites could rescue the inhibitory effect of B28. Thus, a series of metabolites was supplemented in B28 treated cells. We found α-KG, isocitric acid, citric acid, oxalacetic acid and glutamate could partially abolish the reduction of cell viability induced by B28, while supplement of Succinic acid, Malate, Fumaric acid and Glutamine has no significance on cell viability, NAC was used as a positive control (Fig. 5A). Then, we also found the ratio of GSH and GSSG was decreased in B28 treated cell samples (Fig. 5B). Next, we found glutamine transporter SLC1A5/ASCT2, glutaminase 1 isoforms KGA and GAC, glutathione Peroxidase 4 (GPX4) and transcription factor c-Myc were decreased in B28 treated cell samples, while the glutamate dehydrogenase 1 (GLUD1) was not affected (Fig. 5C). Simultaneously, the mRNA levels of SLC1A5, GLS, MYC, GPX4 and SLC3A2 were also not significantly changed (Additional file 8: Fig. S6A). Combined with the data in Fig. 5A, we speculated that KGA/GAC could be a downstream target of B28. To realize this hypothesis, GLS1 was depleted in PANC-1 and CFPAC-1 cells. We found relative cell viability and GSH/GSSG were both reduced, while total ROS level was increased in GLS1 knockdown cells (Figs. 5D and 6E, F). Then, we further confirmed that NAC could partially rescue reduction of cell growth in GLS1 knockdown cells (Fig. 5G, H). Furthermore, we validated that GLS1 knockdown decreased overall OCR and EACR in PAAD cells, and this effect could be partially abolished by NAC (Fig. 5I and Additional file 8: Fig. S6B). We also found reduction of maximal respiration, basal glycolysis and glycolytic reverse in GLS1 knockdown cells were recovered by NAC (Additional file 8: Fig. S6C, D). These data indicated that down-regulation of GLS1 probably contributes to tumor suppressive activity of B28.

### B28 regulates GLS1 expression by modulating YTHDF1

As mentioned above, the protein level of KGA/GAC was decreased while the mRNA remained unchanged. Importantly, this reduction of KGA/GAC was not due to protease-dependent degradation through MG132 addition, indicating a potential mechanism of mRNA stability or translation (Additional file 8: Fig. S7A). Therefore, mRNA levels of m6A associated molecules in vehicle and B28 treated cells were analyzed and no significance was found (Additional file 8: Fig. S7B). Recent study demonstrated YTHDF1 promoted protein synthesis of GLS1 which was dependent on the m6A modification of GLS1 [22]. Similarly, we found YTHDF1 was significantly decreased in B28 treated cells (Fig. 6A). To further understand the potential role of YTHDF1 in regulating glutamine metabolism, we analyzed indicated molecules and found c-Myc, KGA/GAC were both down-regulated in YTHDF1 knockdown cells (Fig. 6B). Simultaneously, data showed cell viability was decreased while total ROS level was increased in YTHDF1 depleted cells (Fig. 6C). We also found that knockdown of YTHDF1 inhibited overall OCR and ECAR (Fig. 6D, E). Consistently, basal respiration, maximal respiration, ATP production (OCR), basal glycolysis, maximal glycolysis and glycolytic reverse were all decreased due to YTHDF1 knockdown (Additional file 8: Fig. S7C). Next, we found overexpression of YTHDF1 could attenuate the inhibitory effect of B28 on cell viability (Fig. 6F). Meanwhile, B28 induced cell apoptosis was also partially abolished in YTHDF1 overexpressed cells (Fig. 6G). We also found reduction of overall OCR and ECAR as well as indicated indexes was recovered due to YTHDF1 overexpression (Fig. 6H, I, Additional file 8: Fig. S7D).

### B28 exhibits potent tumor-suppressive activity in vivo

**Fig. 7:**
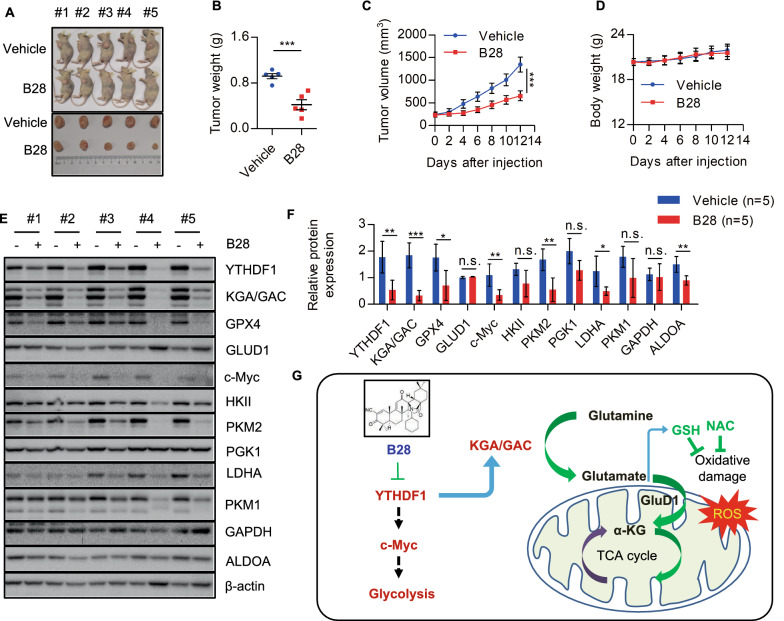
B28 inhibits PAAD cell growth in vivo. **A** Nude mice and tumor sections images of vehicle and B28 administrated groups. **B** Quantitative analysis data of tumor weight. **C** Tumor growth curve of vehicle and B28 administrated groups. **D** Body weight of nude mice in indicated groups. **E** Tumor tissues were subjected to western blot analysis with indicated antibodies. **F** Gray quantitative data of indicated molecules in **E**. **F** Model diagram of B28 inhibits PAAD via regulating YTHDF1-GLS1 axis

To further confirm the tumor suppressive properties of B28, xenograft nude mice assay was performed to evaluate anti-tumor activity of B28 in vivo. Data showed tumor size was reduced with B28 administration (Fig. 7A). We also analyzed tumor weight of tumor section and found tumor weight was reduced in B28 treated group (Fig. 7B). Consistently, data of tumor growth curve showed cell growth was significantly depressed with B28 administration (Fig. 7C). To further evaluate toxic side effect of B28, body weight was assessed and no significance was found between two groups (Fig. 7D). Finally, we found YTHDF1, KGA/GAC, GPX4, c-Myc, and PKM2 were significantly decreased in B28 treated samples which was consistent with the data in vitro (Fig. 7E). Collectively, these data suggest promising anti-tumor activity of this derivative in PAAD.

## Discussion

In this study, anti-tumor activity of B28 was tested. We confirmed that B28 significantly suppressed PAAD cell growth in vitro and in vivo, which was consistent with our previous data. Importantly, we showed a large number of biological processes including cell metabolism pathways were significantly changed in vehicle and B28 treated PAAD cells.

Previous studies have showed that metabolic reprogramming is a key hallmark of cancer cells and targeting cell metabolism could be considered as a novel strategy for cancer therapy [21]. Here, we showed B28 repressed cellular bioenergetics through down-regulating certain subunits of ETC and glycolytic enzymes, interrupting mitochondrial dynamics. Mitochondria are the centers of ATP generation and multiple biological processes such as OXPHOS, TCA cycle, fatty acid metabolism, glutamine metabolism and monocarboxylic acid metabolism [23]. Therefore, mitochondria homeostasis is crucial for carcinogenesis and progression. Multiple reviews have summarized and highlighted targeting mitochondria and mitochondrial metabolism could be considered as a promising strategy for cancer therapy [24, 25]. Additionally, we found B28 induced mitochondrial depolarization, fragment and repressed mitochondrial dynamics by decreasing DRP1 and MFN1. Maintenance of mitochondrial membrane potential is indispensable for mitochondrial homeostasis and loss of mitochondrial membrane potential is considered as a crucial event of cell apoptosis [26]. It has been well validated that OPA1 was important for cristae remodeling and mitochondrial organization, mutations or abnormal cleavage of OPA1 would induce mitochondrial dysfunction [27–29]. We suggest that B28 induced OPA1 cleavage probably due to loss of mitochondrial membrane potential which was consistent with the previous study [27]. Importantly, still there remains a question that whether reduction of respiratory subunits caused by B28 contributes to loss of mitochondrial membrane potential remains to be clarified and further work will focus on addressing this point. Additionally, we also found aerobic glycolysis was depressed in B28 treated cells due to reduction of glycolysis catalytic enzymes as PKM2, LDHA and ENO1. Similarly, recent study has demonstrated that oleanane triterpenoid CDDO-Me could bind and inhibit pyruvate kinase M2 [30]. Thus, we speculated that B28 acts as an oleanane triterpenoid derivative may retain this activity.

It was well validated that mitochondria are the major source of ROS generation, and excessive ROS would lead to mitochondrial DNA mutation, lipid peroxidation, abnormal energy and metabolism, organelle damage, cell damage and apoptosis [31]. Our data showed total ROS was increased by B28. Additionally, we demonstrated that cell apoptosis and cellular bioenergetics dysfunction induced by B28 could be well abolished by ROS scavenger NAC, indicating that anti-tumor activity of B28 probably dependent on excessive ROS generation. We further found several TCA metabolites as α-KG, isocitric acid, citric acid, oxalacetic acid could partially rescue reduction of cell viability induced by B28. Meanwhile, glutamate, but not glutamine, could significantly protect cells from toxicity of B28. Importantly, the ratio of GSH and GSSG was decreased in B28 treated cells, indicating that glutaminolysis may be prohibited induced by B28. Glutamine is a key precursor substance for GSH synthesis which is crucial for redox homeostasis. Reduction of glutamine uptake and glutaminolysis restricts GSH synthesis which contributes to excessive ROS generation [32]. We found glutamine transport and glutaminolysis catalytic enzymes as SLC1A5 and KGA/GAC were significantly decreased in B28 treated cells. A recent study reported that GLS1 is overexpressed in human pancreatic ductal adenocarcinoma (PDAC) and SUCLA2 mediated succinylation of GLS (K311) could eliminate oxidative stress [33]. Consistently, we demonstrated that GLS1 depletion inhibited cell growth and increased total ROS level. Moreover, overall OCR and ECAR were decreased in GLS1 depleted cells, and this reduction of cellular bioenergetics was also attenuated by NAC. These data indicate that GLS1 probably be a downstream target of B28.

Next, we found the mRNA of GLS1 was not significantly changed in vehicle and B28 treated cell samples. Additionally, proteases inhibitor MG132 could not increase the protein level of KGA/GAC. Therefore, we speculated that mRNA instability or reduction of translation efficiency of *GLS1* may result in decreased protein level of GLS1. A recent study showed that YTHDF1 could regulate mRNA translation of GLS1 to promote cisplatin resistance [22]. In PAAD, YTHDF1 was identified as an independent prognostic factor [34]. Thus, we analyzed mRNA level of m6A associated molecules and no significance was found in vehicle and B28 treated cells. However, protein level of YTHDF1 was dramatically decreased due to B28 addition. We further validated that YTHDF1 depletion downregulated KGA/GAC expression as well as inhibited cell viability and cellular bioenergetics. Importantly, we demonstrated that overexpression of YTHDF1 could well abolish the effects of B28 on cell apoptosis and bioenergetics crisis. These data indicated that B28 down-regulates GLS1 to promote oxidative stress and metabolic crisis probably through modulating YTHDF1. YTHDF1 function as a key m6A reader, can modulate multiple genes translation. We found another important transcription factor c-Myc was also down-regulated in YTHDF1 depleted cells which was consistent with the recent studies [35, 36]. Multiple works have elucidated that c-Myc plays a very important role in promoting metabolic reprogramming [37, 38]. Activation of c-Myc-LDHA axis enhances glycolysis to promote PAAD [39]. Therefore, we speculated that down-regulation of LDHA induced by B28 may be reason to c-Myc depletion. Further work needs to clarify the detailed mechanism about YTHDF1 regulates expression of c-Myc and glycolysis catalytic enzymes.

## Conclusions

In summary, our study evaluated potential anti-tumor activities of a novel Olean- 28,13β-lactam (B28) in PAAD. We demonstrated that B28 promotes excessive ROS to increase oxidative damage which in turn results to cell apoptosis and cellular bioenergetics crisis. Moreover, we verified B28 suppresses YTHDF1-GLS1 axis to promote oxidative stress and metabolic crisis. These findings highlight novel olean-28,13 β-lactam could be considered as a potential chemotherapeutic agent to intervene PAAD progression.

## Supplementary Information

**Additional file 1.** List of up-regulated genes in B28 vs. vehicle samples.

**Additional file 2.** List of down-regulated genes in B28 vs. vehicle samples.

**Additional file 3.** List of KEGG enrich significant genes in B28 vs. vehicle samples.

**Additional file 4.** List of GO enrich significant genes in B28 vs. vehicle samples.

**Additional file 5.** List of Reactome enrich significant genes in B28 vs. vehicle samples.

**Additional file 6: Table S1.** Detail information of antibodies used in this study.

**Additional file 7: Table S2.** Detail information of plasmids used in this study.

**Additional file 8: Figure S1.** Molecular Function (MF), Biological Process (BP) and Cellular Components (CC) analysis vehicle and B28 treated cell samples. (B28 vs. vehicle). **Figure S2.** Enrichment analysis of down-regulated and up-regulated differentially expressed genes (DEGs) based on GO database. (B28 vs. vehicle). **Figure S3.** Enrichment analysis of down-regulated and up-regulated differentially expressed genes (DEGs) based on Reactome database. (B28 vs. vehicle). **Figure S4.** Indexes of OCR and ECAR in B28 and vehicle treated cell samples. **Figure S5.** NAC significantly abolishes the effects of B28 on cell cycle, mitochondrial membrane potential and cellular bioenergetics. **Figure S6.** NAC blocks inhibition of cellular bioenergetics in GLS1 knockdown cells. **Figure S7.** Overexpression of YTHDF1 attenuates the effect of B28 on cellular bioenergetics in PAAD cells.

## Data Availability

The data used in the present study are available from the corresponding author upon reasonable request.

## Abbreviations

DRP1: Dynamin-relatedprotein 1
ECAR: Extracellular acidification rate
ENO1: Enolase 1
ETC: Electron transport chain
GLUD1: Glutamate dehydrogenase 1
GPX4: Glutathione peroxidase 4
GLS1: Glutaminase 1
GO: Gene ontology
hERG: Human Ether-à-go-go-RelatedGene
KEGG: Kyoto Encyclopedia of Genes and Genomes
LDHA: Lactate dehydrogenase A
MFN1: Mitofusin 1
MTCO I: Mitochondrial cytochrome c oxidase subunit I
MTCO II: Mitochondrial cytochrome c oxidase subunit II
MTCO III: Mitochondrial cytochrome c oxidase subunit III
NAC: *N*-Acetyl-l-cysteine
NDUFS6: NADH:ubiquinone oxidoreductase subunit S6
ND1: NADH:ubiquinone oxidoreductase core subunit 1
ND3: NADH:ubiquinone oxidoreductase core subunit 3
OCR: Oxygen consumption rate
OPA1: Optic atrophy 1
OMA1: OMA1 zinc metallopeptidase
PAAD: Pancreatic adenocarcinoma
PPAR-γ: Peroxisome proliferator activated receptor gamma
PKM2: Pyruvate kinase M2
SLC1A5: Solute Carrier Family 1 Member 5
TCA: Tricarboxylic acid cycle
YTHDF1: YTH *N*6-methyladenosine RNA binding protein 1
