# Supplementary material for: A synthesized olean-28,13β-lactam targets YTHDF1-GLS1 axis to induce ROS-dependent metabolic crisis and cell death in pancreatic adenocarcinoma
Source: Cancer Cell Int. 2022 Apr 2;22:143. doi: 10.1186/s12935-022-02562-6 (PMC8976991; doi:10.1186/s12935-022-02562-6)
Supplement: Supplementary file 6 — Additional file 6: Table S1. Detail information of antibodies used in this study. [file 12935_2022_2562_MOESM6_ESM.docx]

**Table S1**

**Detail information of antibodies.**

| **Name** | **Cat.No** | **Company** |
| --- | --- | --- |
| PARP | #9532 | Cell Signaling Technology, Inc. |
| Caspase 3 | #14220 | Cell Signaling Technology, Inc. |
| β-actin | #3700 | Cell Signaling Technology, Inc. |
| MTCO I | ab14705 | Abcam |
| MTCO II | ab110258 | Abcam |
| MTCO IV | ab33985 | Abcam |
| NDUFA5 | 16640-1-AP | [Proteintech Group](https://www.ptgcn.com/) |
| NDUFS6 | #14417-1-AP | [Proteintech Group](https://www.ptgcn.com/) |
| ND1 | ab181848 | Abcam |
| ND3 | ab192306 | Abcam |
| c-Myc | #18583 | Cell Signaling Technology, Inc. |
| PGC-1α | #66369-1-Ig | [Proteintech Group](https://www.ptgcn.com/) |
| PPAR-γ | #16643-1-AP | [Proteintech Group](https://www.ptgcn.com/) |
| OPA1 | 612607 | BD sciences |
| DRP1 | 12957-1-AP | [Proteintech Group](https://www.ptgcn.com/) |
| MFN1 | ab104274 | Abcam |
| OMA1 | 17116-1-AP | [Proteintech Group](https://www.ptgcn.com/) |
| YME1L | 66551-1-Ig | [Proteintech Group](https://www.ptgcn.com/) |
| PKM2 | A19102 | Abclonal |
| LDHA | A0861 | Abclonal |
| ENO1 | #3810 | Cell Signaling Technology, Inc. |
| GCK | A6293 | Abclonal |
| PFKM | ab154804 | Abcam |
| PFKL | A7708 | Abclonal |
| HKII | A0994 | Abclonal |
| GLUT1 | 21829-1-AP | [Proteintech Group](https://www.ptgcn.com/) |
| ALDOA | [A11445](https://abclonal.com.cn/catalog/A11445) | Abclonal |
| PGK1 | 17811-1-AP | [Proteintech Group](https://www.ptgcn.com/) |
| GAPDH | A19056 | Abclonal |
| GPI | A6916 | Abclonal |
| PKM1 | #7067 | Cell Signaling Technology, Inc. |
| Phospho-SAPK/JNK (Thr183/Tyr185) | #9255 | Cell Signaling Technology, Inc. |
| SLC1A5 | 20350-1-AP | [Proteintech Group](https://www.ptgcn.com/) |
| KGA/GAC | 12855-1-AP | [Proteintech Group](https://www.ptgcn.com/) |
| GLUD1 | 14299-1-AP | [Proteintech Group](https://www.ptgcn.com/) |
| GPX4 | A11243 | Abclonal |
| YTHDF1 | 17479-1-AP | [Proteintech Group](https://www.ptgcn.com/) |
| HRP-labeled goat anti rabbit secondary antibody | A0208 | Beyotime Beyotime Biotech |
| HRP-labeled goat anti mouse secondary antibody | A0216 | Beyotime Beyotime Biotech |
