# Supplementary material for: A synthesized olean-28,13β-lactam targets YTHDF1-GLS1 axis to induce ROS-dependent metabolic crisis and cell death in pancreatic adenocarcinoma
Source: Cancer Cell Int. 2022 Apr 2;22:143. doi: 10.1186/s12935-022-02562-6 (PMC8976991; doi:10.1186/s12935-022-02562-6)
Supplement: Supplementary file 7 — Additional file 7: Table S2. Detail information of plasmids used in this study. [file 12935_2022_2562_MOESM7_ESM.docx]

**Table S2**

Detail information of plasmids.

Plasmids used in this study were purchased from PPL (Public Protein/Plasmid Library).

| **Information list** | **GLS1 shRNA** | **YTHDF1 shRNA** | **YTHDF1 OE** |
| --- | --- | --- | --- |
| Cat.No. | 2744 | 54915 | BC050284 |
| Vector Backbone | pPLK GFP+Puro | pPLK GFP+Puro | pLenti-CMV-GFP-puro |
| **Vector type** | Lentiviral interference | Lentiviral interference | Lentiviral expression |
| **Cloning site 5'** | BamH I | BamH I | None |
| **Cloning site 3'** | EcoR I | EcoR I | None |
| **Bacterial resistance(s)** | Ampicillin | Ampicillin | Ampicillin |
| **Selectable markers** | Puromycin | Puromycin | Puromycin |
