# Supplementary material for: A synthesized olean-28,13β-lactam targets YTHDF1-GLS1 axis to induce ROS-dependent metabolic crisis and cell death in pancreatic adenocarcinoma
Source: Cancer Cell Int. 2022 Apr 2;22:143. doi: 10.1186/s12935-022-02562-6 (PMC8976991; doi:10.1186/s12935-022-02562-6)
Supplement: Supplementary file 8 — Additional file 8: Figure S1. Molecular Function (MF), Biological Process (BP) and Cellular Components (CC) analysis vehicle and B28 treated cell samples. (B28 vs. vehicle). Figure S2. Enrichment analysis of down-regulated and up-regulated differentially expressed genes (DEGs) based on GO database. (B28 vs. vehicle). Figure S3. Enrichment analysis of down-regulated and up-regulated differentially expressed genes (DEGs) based on Reactome database. (B28 vs. vehicle). Figure S4. Indexes of OCR and ECAR in B28 and vehicle treated cell samples. Figure S5. NAC significantly abolishes the effects of B28 on cell cycle, mitochondrial membrane potential and cellular bioenergetics. Figure S6. NAC blocks inhibition of cellular bioenergetics in GLS1 knockdown cells. Figure S7. Overexpression of YTHDF1 attenuates the effect of B28 on cellular bioenergetics in PAAD cells. [file 12935_2022_2562_MOESM8_ESM.docx]

**A Synthesized Olean-28,13β-lactam targets YTHDF1-GLS1 axis to induce ROS-dependent metabolic crisis and cell death in pancreatic adenocarcinoma**

Shijia Wu^1†^, Yong Ai^2,3,4†^, Huimin Huang^5†^, Guangyu Wu^6^, Shipeng Zhou^1^, Weilong Hong^1^, Percy David Papa Akuetteh^1^, Guihua Jin^1^, Xingling Zhao^1^, Yihua Zhang^3,4^*, Xiaolong Zhang^7^*, Linhua Lan^1^*

^1^Key Laboratory of Diagnosis and Treatment of Severe Hepato-Pancreatic Diseases of Zhejiang Province, The First Affiliated Hospital of Wenzhou Medical University, Wenzhou 325000, P.R.China.

^2^ Department of Pharmacology, University of Maryland, Baltimore, United States.

^3^ State Key Laboratory of Natural Medicines, China Pharmaceutical University, Nanjing 210009, P. R. China

^4^Jiangsu Key Laboratory of Drug Discovery for Metabolic Diseases, China Pharmaceutical University, Nanjing 210009, P.R.China.

^5^School of Laboratory Medicine and Life Sciences, Wenzhou Medical University, Wenzhou 325000, P.R. China.

^6^Department of Trauma Surgery, The First Affiliated Hospital of Wenzhou Medical University, Wenzhou 325000, P.R.China.

^7^Department of Anesthesiology, Critical Care and Pain Medicine, The Second Affiliated Hospital and Yuying Children's Hospital of WenZhou Medical University, 109 Xueyuan Western Road, Wenzhou, Zhejiang Province, 325027, P.R.China.

^†^Contributed equally.

***Address correspondence to***: Dr. Linhua Lan, e-mail: [*paullee90@wmu.edu.cn*](mailto:paullee90@wmu.edu.cn); Dr. Xiaolong Zhang, email: [*zxl307@126.com*](mailto:zxl307@126.com); Prof. Yihua Zhang, email: [*zyhtgd@163.com*](mailto:zyhtgd@163.com).

**Running title**: Targeting YTHDF1-GLS1 axis to suppress PAAD.

**Keywords:** Oleanolic acid; metabolic crisis; glutaminolysis; YTHDF1; redox homeostasis

**Figure S1**

**Legend:** Molecular Function (MF), Biological Process (BP) and Cellular Components (CC) analysis vehicle and B28 treated cell samples. (B28 VS vehicle)

**Figure S2**

**Legend:** Enrichment analysis of down-regulated and up-regulated differentially expressed genes (DEGs) based on GO database. (B28 VS vehicle)

**Figure S3**

**Legend:** Enrichment analysis of down-regulated and up-regulated differentially expressed genes (DEGs) based on Reactome database. (B28 VS vehicle)

**Figure S4**

**Legend:** A Analysis of basal respiration, maximal respiration and ATP production associated OCR in vehicle and B28 treated cells. B Analysis of spare respiratory capacity, proton leak and non-mito oxygen consumption in in vehicle and B28 treated cells. C Analysis of basal glycolysis, glycolytic capacity and glycolytic reverse in vehicle and B28 treated cells.

**Figure S5**

**Legend:** A Cell cycle distribution of vehicle, B28 and B28+NAC treated cells. B Analysis of MMP in indicated cell groups. C Analysis of basal respiration, maximal respiration and ATP production associated OCR in vehicle, B28 and B28+NAC treated cells. D Analysis of basal glycolysis, glycolytic capacity and glycolytic reverse in vehicle, B28 and B28+NAC treated cells.

**Figure S6**

**Legend:** A mRNA level of SLC1A5, GLS1, MYC, GLUD1, GPX4 and SLC3A2 in vehicle and B28 treated cell samples. B Overall OCR and ECAR curves of shCont, shGLS1#1 and shGLS1#1+NAC CFPAC-1 cells. C Analysis of basal respiration, maximal respiration and ATP production associated OCR in indicated cell groups. D Basal glycolysis, glycolysis capacity and glycolytic reverse in shCont, shGLS1#1 and shGLS1#1+NAC cells.

**Figure S7**

**Legend:** A Protein and mRNA level of KGA/GAC in indicated cell groups. B mRNA level of m6A associated genes in vehicle and B28 treated PANC-1 cells. C Multiple indexes of OCR and ECAR in control and YTHDF1 knockdown cells. D Multiple indexes of OCR and ECAR in CMV+vehicle, CMV+B28, YTHDF1 OE+vehicle and YTHDF1+B28 cell groups.
